# ”Why should I pay for this vaccine?” – Norwegian adolescents views on meningococcal vaccination

**DOI:** 10.1186/s12889-026-27591-y

**Published:** 2026-04-30

**Authors:** Rebecca Nybru Gleditsch, Sara Viksmoen Watle, Bo T. Hansen, Lisbeth M. Naess, Lene Kristine Juvet, Trine Skogset Ofitserova

**Affiliations:** 1 Fafo Institute for Labour and Social Research, PO Box 2947, Tøyen, Oslo, NO-0608 Norway; 2 Norwegian Institute of Public Health, PO Box 222, Skøyen, Oslo, N-0213 Norway; 3 Norwegian Medical Products Agency, PO Box 240, Skøyen, Oslo, N-0213 Norway; 4 Division of Infection Control, Norwegian Institute of Public Health, PO Box 222, Skøyen, Oslo, N-0213 Norway

**Keywords:** Adolescent Health, Vaccine-Preventable Diseases, Meningococcal Vaccines, Meningitis, Health Inequities, Immunization Programs, Thematic analysis, Focus groups, Qualitative research

## Abstract

**Background:**

As one of few countries in Europe, Norway does not offer vaccination against invasive meningococcal disease (IMD) as part of the National Immunization Program for Children (NIP). Instead, adolescents aged 16–19 are recommended to consider having the meningococcal ACWY conjugate vaccine (MCV4) if they participate in activities that increase the risk of IMD. Meningococcal vaccination rates in Norway are low compared to vaccines offered through the NIP. Whereas all vaccinations in the NIP are free and organized through schools or municipal health clinics, there is no uniform system in place for the meningococcal vaccine. On the contrary, both administration practices and cost of vaccination vary nationally, with some counties offering the vaccine for free, while others offer the vaccine at a cost of $40–80. The aim of this study was to explore adolescents’ awareness, knowledge, and decision-making processes related to meningococcal disease and vaccination.

**Methods:**

Using a semi-structured interview guide, we conducted 18 focus group interviews in the fall of 2022, involving a total of 126 high school seniors in Norway. The participants were asked to share their views, knowledge and experiences regarding invasive meningococcal disease and vaccination. We used NVivo 14 to facilitate the thematic analysis and a codebook analysis approach was conducted to identify themes and patterns in the data.

**Results:**

The analysis identified three central themes emerging from the discussions with the adolescents. These emphasize how information, cost, and location and guidance impact the adolescents’ decision-making and ability to get vaccinated.

**Conclusions:**

The current organization of MCV4 vaccination in Norway do not meet the needs identified by the adolescents in this study. This results in unequal access to a highly effective vaccine that protects healthy adolescents against a deadly disease. The present study highlights the need to address vaccination disparities and accessibility, as well as increasing awareness and knowledge about meningococcal disease and vaccination to ensure equal access for all adolescents.

**Supplementary Information:**

The online version contains supplementary material available at 10.1186/s12889-026-27591-y.

## Background

Invasive meningococcal disease (IMD) is caused by the bacterium *Neisseria meningitidis*, or the meningococcus, which is a common commensal in the human upper respiratory tract. The disease is severe with high mortality and morbidity, and a fatality rate of 8–15% [1]. Many countries have a peak in IMD among adolescents, including Norway, and previous research has linked this to high transmission rates, social activities and lifestyle changes among adolescents [2, 3]. Because of its severity, meningococcal vaccines are offered through organized programs in several countries, including 18 European countries [4]. Countries with organized adolescent meningococcal vaccination have experienced a substantial decrease in IMD and the World Health Organization (WHO) has made a call for action to defeat IMD by 2030 [5]. Despite this, Norway does not include any type of meningococcal vaccine in the National Immunization Program for children (NIP) [6].

For many years, Norway has been among the countries with the highest coverage in the NIP, with a national coverage of 90–94% for 16-year-olds in 2023 [7]. There is a high level of confidence in childhood vaccination in Norway [8]. The program is voluntary and free of charge and currently includes vaccines against twelve infectious diseases [6]. Although no type of meningococcal vaccine is included in the NIP, the Norwegian Institute of Public Health (NIPH) has recommended 16–19 year-olds to consider vaccination on an individual basis since 2011 [9]. In Norway, adolescents gain legal autonomy in health care matters when they turn 16 and as a general rule, parents no longer have access to their healthcare information. The MCV4 vaccine is the first vaccine adolescents in Norway are recommended that is not part of the NIP and this is also the first vaccine recommended after turning 16, when they alone are required to consent to their own vaccination.

The recommendation followed an upsurge in IMD cases associated with the so-called “russ celebration”, which is a Norwegian rite of passage among high school seniors (age 18–19 years) who are referred to as “russ” [9, 10]. The most dedicated russ spend a lot of money on renovating old buses into rolling nightclubs, which is particularly common in the greater Oslo area, and often quite expensive [10]. The most recent russ cohort from 2024 estimates spending up to $200,000 (2 million NOK) per russ bus [11]. Although the parties most often involve the current russ, younger high school students may sometimes be invited. The weeks of russ celebration often involve close contact between the adolescents, partying, binge drinking, sharing bottles, active or passive smoking, hooking up, as well as lack of sleep. Over the last 10 years, most cases of IMD in Norway have been associated with the russ celebration and those planning to participate have been specifically recommended to get the vaccine [12], which has become known to many as the “russ vaccine”. Although the russ celebration officially starts in the beginning of May, many adolescents will party extensively with their peers throughout their senior year.

**Fig. 1:**
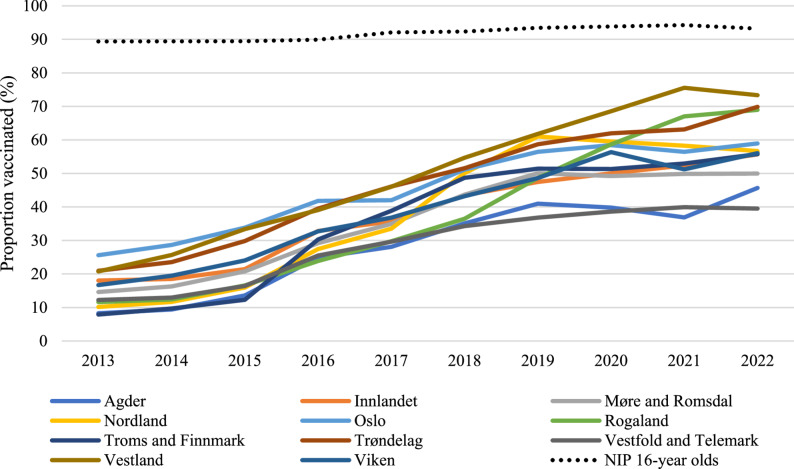
Vaccination coverage in Norway 2013-2022. MCV4 among 18-19 year-olds, by county (solid lines) and national coverage in the NIP for children at 16 years of age (dashed line) (Norwegian immunization registry, SYSVAK)

However, as meningococcal vaccine is not part of the NIP there are no national guidelines for funding or administration of these vaccines. This results in great variation throughout the country in how the vaccine is distributed and the cost of vaccination, with certain regions fully funding the vaccines for all seniors in high school and other municipalities offering vaccines at a cost of approximately $40–80 [13]. After MCV4 vaccination was recommended for teenagers in 2011, the coverage of MCV4 has gradually increased (Fig. 1). As the recommendation specifically mentions the russ celebration and most adolescents are offered the vaccine during their senior year, the uptake is highest among 18-19-year-olds. National coverage of MCV4 in this age group increased from 18% in 2013 to 59% in 2022 (Fig. 1). This rise is likely attributable to an increasing number of counties sponsoring the vaccine during this period, as well as increased awareness driven by school health services and media coverage of reported cases. However, coverage of the MCV4 is far below the coverage of vaccines included in the NIP and it also shows large regional variation (Fig. 1).

Adolescent vaccination is a relatively understudied topic and a recent systematic review highlighted adolescents’ views on vaccines to be particularly underrepresented in qualitative research [14]. The review further showed that the majority of the qualitative studies on adolescents’ experiences with vaccine decisions focuses on human papilloma virus vaccines (HPV) and that more qualitative research examining vaccines other than the HPV vaccine is needed [14]. A recent study also suggested that improving vaccine uptake among adolescents should involve the adolescents and informing them about vaccine safety and effectiveness [15].

The international literature is also scarce regarding adolescent meningococcal vaccine uptake [16]. Previous studies indicate a knowledge gap among teenagers, parents and healthcare providers regarding both IMD and available vaccines [17–19]. Furthermore, adolescents’ decision making when offered meningococcal vaccination has been found to be impacted by knowledge and beliefs about vaccination and or disease [20–23], important people around them such as parents [20–22, 24], the process of decision [24], trust in vaccination [20, 21, 23, 24], vaccination location [25, 26], as well as social responsibility [22].

While we have information regarding variations in MCV4 vaccination coverage in Norway at regional level, little is known about why the coverage varies geographically. Even less research has focused on knowledge about meningococcal vaccines and IMD among adolescents in the target group. We know that providing adolescents with enough information about vaccination is key to ensure that they can make informed health choices [14, 25]. To our knowledge, no previous research has explored Norwegian adolescents’ awareness, knowledge, and decision-making processes related to meningococcal disease and vaccination, and the current study aims to address this.

## Methods

### Study design

A qualitative research design was used to explore the views and experiences of adolescents in Norway. In the fall of 2022, 18 focus group interviews were conducted with a total of 126 high school seniors at five different schools. These had been purposefully selected based on previous research finding geographical differences in who participates in the “russ celebration” [27] and social behavior that increase transmission risk [12]. We aimed to include schools in the Western parts of Oslo with a higher share of adolescents typically participating in the russ celebration, as well as schools located in the Eastern parts of Oslo where fewer adolescents typically participate in the celebration [10]. Participants were recruited through their high school. The schools were approached by phone and follow-up emails. In total, 11 schools were contacted (8 in Oslo, 3 in Viken) and in-person information meetings with the adolescents were held at schools that agreed to participate (5 in total). Although a few of the students had been vaccinated prior to the interview, none of the students had been offered the MCV4 vaccine at their high school at the time of the interviews but would receive an offer of the vaccine the following January or February. The adolescents received information about the study and informed consent through the school’s digital communication platform and/or from their teachers prior to the meetings. The first phase of the recruitment focused on schools in Oslo, but due to schools declining to participate (the interviews were conducted shortly after an extensive teacher strike [28], which several schools cited as the main reason for declining to participate), recruitment was extended to the neighboring region of Viken to increase the share of adolescents planning on taking part in the russ celebration. Recruitment of new participants continued until data saturation was reached, defined as the point at which no new themes emerged in the interviews [29].

### Focus groups

Focus groups were chosen as our data collection method to generate discussion about adolescents’ awareness, knowledge and decision-making process related to meningococcal disease and vaccination, allowing the participants to build on, challenge, and reflect on each other’s perspectives. We deemed this method as particularly beneficial as this is an understudied topic in the Norwegian context and the group interaction can surface shared norms as well as differing viewpoints that may not emerge in individual interviews. Prior to each interview, the informed consent to study participation was explained orally to the group of adolescents. All adolescents who agreed to participate signed an informed consent form. As all participants were 16 years or older, no parent consent was needed. Each participant also completed a questionnaire with five demographic questions (results shown in Table 1). Two moderators conducted the interviews in a private room following a semi-structured interview guide [30], designed in collaboration with researchers and physicians working with meningococcal vaccination and IMD surveillance at the NIPH. To moderate potential peer pressure, the groups were facilitated by the moderators to encourage respectful dialogue, explaining that there were no right or wrong answers, and ensure that all perspectives were welcomed. As no past research on Norwegian adolescents’ views and knowledge about meningococcal vaccine and disease were identified prior to this study, we utilized themes emerging from past qualitative studies on adolescents and vaccination uptake in other countries [14, 17–19, 31], to structure the interview guide and create open-ended questions [30]. Topics used to structure the guide were awareness and knowledge about the vaccine and IMD, process of vaccination decisions, and trusted sources of information. The semi-structured interview guide ensured that the same topics were discussed in all focus groups, while also having flexibility to adapt and go along with the direction of the discussions [30].

**Table 1:** Overview of participants (*n* = 126)

		*n* (%)
Gender	Male	38 (30)
	Female Did not reply	87 (69) 1 (1)
Adolescents’ residence	Oslo, East	27 (21)
	Oslo, West	34 (27)
	Viken, Municipality A	28 (22)
	Viken, Municipality B	37 (29)
High school location	Eastern part of Oslo Western part of Oslo Viken	32 (25) 31 (25) 63 (50)
Days partying per month	0	20 (16)
	1	10 (8)
	2	8 (6)
	3	16 (13)
	4	32 (25)
	5+	40 (32)
Participation in russ celebration	Yes, highly involved^a^	69 (55)
	Yes^b^	31 (25)
	No	16 (12)
	Undecided	10 (8)

^a^ “Yes, highly involved” is defined as going to be part of a nightclub russ bus

^b^ “Yes” is defined as planning on participating in the russ celebration, but not part of a nightclub russ bus

### Data analysis

Semi-structured focus group interviews were planned, structured, developed, conducted, and analyzed according to the method of Kreuger and Casey [30]. Special attention was paid to develop good and active questions that aimed to create discussion among the participants, as well as using the introduction to the focus group to create a thoughtful, informal and welcoming atmosphere for the discussion [30]. Focus group recordings were transcribed manually by one of the interviewers and checked for accuracy by the other interviewer. All focus group transcripts were imported into the Qualitative Solutions and Research International NVivo software program 14 to facilitate the thematic process. A thematic analysis using a codebook analysis approach [32] was conducted to identify themes and patterns related to the adolescents’ awareness, knowledge and decision-making processes. The codebook was developed following the guidelines of Boyatzis [33] and included code label, definition, description, qualifications, exclusions, and examples [33]. The codebook was utilized to guide the thematic analysis and helped ensure coding accuracy and intercoder agreement among the team members involved in the data analysis (TSO and RNG) [34]. First, the data was independently coded in NVivo by each researcher. Then, the initial codes were compared and discussed to reach a consensus on the final version of the codebook. Overall, there was a consistent agreement on the coding. The final version was used to guide the analysis of the remaining transcripts and any new codes that were identified were first discussed among the researchers before added to the codebook.

## Results

### Study population

Adolescents who wanted to participate were initially grouped into focus groups consisting of 6–8 participants. However, as some focus groups had adolescents not showing up, while others had more showing up than planned, the end results were group sizes ranging from 1 to 14 (median = 7). One scheduled focus group ended up with only one participant, which we included in the data analysis. Among the participants, more than two-thirds identified as female (Table 1). Half of the participants lived in Oslo and the remaining half in Viken. As meningococcal vaccination in Norway is not part of an immunization program, there is regional variation in vaccination cost. Oslo offers MCV4 vaccination free of charge to all high school seniors. However, in Viken, cost varies by municipality. Among participants from Viken, 28 resided in municipality A, which offered free MCV4 vaccination, while 37 resided in municipality B, where individual MCV4 vaccination has an out-of-pocket cost of approximately $60. Viken participants from either municipality attended the same school. More than half of the participants reported partying at least 4 days a month, while 16% never partied (Table 1). The majority of the participants (80%) reported planning to take part in the russ celebration, with 69% of them planning to be highly involved (defined as being part of a nightclub russ bus). Most of the adolescents who were not participating in the russ celebration (or who were undecided) attended schools in the Eastern part of Oslo. With few exceptions, all adolescents attending high schools located in either Viken or the western part of Oslo were planning to participate in the celebration.

### Themes

Throughout the focus groups it became evident that vaccines were not a common topic of conversation among the adolescents. Although most mentioned how they had discussed vaccines with their friends during the COVID-19 pandemic, vaccines were only a topic of conversation when they were offered a vaccine, such as in middle school when they were offered the HPV-vaccine as part of the NIP. For most of the adolescents, vaccination had been something that happened to them as a result of a decision made by someone else, usually their parents. They were rarely the ones that received information about vaccines, and they did not necessarily know what the vaccines were for or how they work, they just showed up and received the vaccine from the school nurse:

*«It’s like in primary school where you did not necessarily know what most of it was*,* you just show up and take it [the vaccine] and then you are done.» (*Male, high school Western Oslo)

Most of the adolescents described vaccination as something most people do. Several viewed vaccination as an important choice, to protect both yourself and society against disease. Furthermore, it seemed as if the lack of discussion around vaccination reflected that this is simply a normal part of their lives:

*«In general*,* almost everyone agrees that vaccines are good*,* there is not a lot of disagreement between people about whether it is good or not. »* (Male, high school Western Oslo)

However, the conversations revealed that for many of the adolescents, the MCV4 differs from other vaccines. Except from the COVID-19 vaccine, the MCV4 is the first vaccine adolescents in Norway are recommended outside the NIP and where their age (above 16) are required to consent to their own vaccination. Through the thematic analysis, three main themes became apparent in the adolescent’s MCV4 vaccine decision process: (1) awareness; (2) cost; (3) location and guidance.

### Theme 1: awareness

#### “What vaccine?”

There was great variation in the adolescents’ awareness and knowledge about meningococcal disease and vaccination. In some focus groups all participants had heard about the vaccine and the disease prior to the focus group, while in others no participants had heard about IMD. It also seemed that those who were planning on being highly involved in russ celebration had heard more about the vaccine compared to those planning to be less involved. However, regardless of how much they knew, almost none of the adolescents we spoke to felt that they knew enough. A majority of them had heard that IMD was very dangerous, such as this adolescent:

*«I could be wrong about this*,* but it is very deadly if you get it*,* you have a very high chance of dying*,* I don’t remember how high.» (*Male, high school Western Oslo)

There were also a handful of adolescents who demonstrated somewhat greater knowledge about IMD:

*«I know it’s very dangerous. And that there’s probably something with the membrane that surrounds the brain*,* that it fills up or inflates or something like that. I don’t really know. And it’s very contagious and it spreads through sharing bottles*,* food*,* cutlery and things like that. And then I remember that a few years ago there was someone in secondary school*,* not my secondary school*,* but a secondary school nearby*,* who died from it. Who was in eighth or ninth grade.» (*Female, high school Viken)

Half of the focus groups (9/18) had one or several participants who knew of someone affected by IMD. Those who had knowledge about someone who got sick and/or died from IMD also seemed to have received more information about vaccines and disease from school and/or parents, as illustrated by this adolescent:

*«I remember we got quite a bit of information at our secondary school because there was someone who died of meningitis.» (*Female, high school Viken)

Throughout our interviews a pattern seemed to be that the adolescents who had more knowledge about IMD viewed the vaccine as more relevant and getting vaccinated as more important, compared to the adolescents we spoke to who had little or no knowledge about IMD

#### “We didn’t know much about it and therefore it wasn’t as important”

Among participants from schools in Western Oslo and Viken, who were more likely to plan on participating in the russ celebration compared to those attending schools in Eastern Oslo, many knew that meningococci are transmitted through saliva or spit. Fewer of the adolescents from schools in Eastern Oslo knew this. Awareness about the vaccine itself was also lower among this group. For instance, among 3 of 5 focus groups from schools in Eastern Oslo, none of the participants had heard about the disease or the vaccine. Overall, the majority of participants from these schools were unaware that they were being offered a vaccine in their senior year of high school and stated that they had not received information about the vaccine or disease from their schools. Among the schools in Western Oslo and Viken, most had heard about the disease by name and knew about the upcoming vaccination but had not received information about the disease or the risk of being unvaccinated. The information they had received were in most cases practical information on how they could get the vaccine:

*«…I don’t feel like we have received information about what the disease is or what the vaccine does. It’s more like ‘you can take the vaccine’.»* (Female, high school Western Oslo)

Adolescents who were familiar with the MCV4 vaccine usually referred to it as the vaccine against meningitis or the “russ vaccine”. The strong association between the MCV4 vaccine and the graduation celebration was evident in the majority of interviews. Most of the adolescents who had heard about the vaccine, associated it with the graduation celebration and many described being told it was especially important to get vaccinated prior to being russ. However, among those who were less aware of the vaccine prior to the interviews, lack of information seemed to have made several of them assume that the vaccine was not relevant for them. This was especially apparent among those who were not going to be involved in the russ celebration and who had heard about the vaccine solely in association with the celebration. Such as this student describing how she thinks youth like herself responds to the description of “russ vaccine”:

*«I think that they would say ‘okay*,* I’m not going to be drunk*,* so then I don’t have to take it.’ [and] ‘I’m not going to party*,* so I’m not going to get the vaccine’.»* (Female, high school Eastern Oslo)

At the end of the interviews, focus groups with low awareness of meningococcal disease and vaccine were provided a short overview of the topic. Learning more about the vaccine and the disease seemed to both enlighten them and make them aware that they need this type of information to be able to make an informed decision. Some described the importance of information in order to decide whether the vaccine was relevant on an individual basis, while others argued that this is not only important for themselves, but for all adolescents in order to be able to make an informed choice about MCV4 vaccination:

*«After this conversation*,* I really feel that the information we have received now [during the interview]*,* everyone should know the same as we now know. Because it’s really important that you are aware of your surroundings*,* especially during the time of the russ celebration. That you are aware that you might get infected by just sharing a drink with someone*,* even if you don’t think about it. And that you can get a vaccine that prevents it.»* Female, high school Eastern Oslo

However, getting enough information about the MCV4 vaccine and disease to decide whether to vaccinate was also important among the adolescents who were already vaccinated or planning to get the vaccine. Many of them explained how lack of information about the vaccine when they were offered the vaccine during their first or second year of high school had impacted their decision:

*“When the offer came in first grade*,* I didn’t know what the vaccine was about. So*,* I basically declined the offer at first and then I got it later. So it would have been good to get information earlier on.» (*Male, high school Viken)

Several of the adolescents told us that they would have made a different decision if they had received information about IMD and the vaccine during their first year of high school. They had been partying with former russ from the year they started high school, but the lack of information made them unable to properly consider the offer at the time, as this student explains:

*«It might have been good to know that it is something you should take into account if you intend to take part in “rulling” [partying in the nightclub russ bus] in the previous years*,* because I took it last year*,* but then I took it too late because there was something about it taking time before it works. So if I had known this earlier then I would have taken it earlier.»* (Female, high school Western Oslo)

The adolescents we talked to, regardless of level of knowledge about the vaccine, disease or involvement in the russ celebration, were all in agreement that they want to be educated about the MCV4 vaccine and disease, and that they viewed information as key to decisions about vaccination.

### Theme 2: cost

As the interviews progressed issues surrounding cost of vaccination became an important topic in the conversations and was frequently seen as the most important barrier to vaccination among the adolescents

“Why is it not free?”

At the high schools in Oslo, where the vaccine was provided for free, most were unaware that seniors in other parts of Norway had to pay for MCV4 vaccination. In Viken, where the cost of the vaccine varied, all participants seemed aware of the financial divide across municipalities. The differences in cost associated with the vaccine was questioned by the participants. They found it unfair that not all adolescents in Norway receive the MCV4 vaccine for free and that it depended on where they live:

*«I think that either everyone needs to get it for free in their senior year or nobody gets it for free. Why should one city in Norway get it*,* while the others don’t? It doesn’t quite make sense.» (*Female, high school Eastern Oslo)

Many of the adolescents emphasized that everyone can potentially get infected with IMD regardless of where they live and this shouldn’t impact the cost of vaccination. Some also found it difficult to understand how the vaccine can cost money, arguing that Norway is supposed to have free and universal health care. Several participants highlighted that not all adolescents necessarily can afford to pay for the vaccine, which could influence whether they were able to get the vaccine. Cost was especially seen as a barrier if they were not provided with enough information about IMD, as explained by this adolescent:

*«That’s what’s scary*,* because those it concerns*,* those for whom it might be too expensive… [they] might not have enough knowledge about the vaccine and about the disease to understand how serious it is. And therefore they think like “this costs a lot of money*,* we can’t afford this*,* and this disease is probably not very serious’*,* perhaps they compare it to the coronavirus*,* which in a way is not so dangerous. And then it [the price] matters a lot in their decision to skip the vaccine*,* or not wanting or not pay for the vaccine for their children.» (*Female, high school Viken)

When asked whether they or adolescents their age would be willing to pay for the vaccine using their own money, the vast majority said no, as illustrated by this adolescent:

*«I know that if I had to pay for it and I could choose for myself whether I wanted to take it or not*,* then I would have said no.» (*Female, high school Eastern Oslo)

Although the vast majority of the participants viewed the price of the vaccine as a barrier to vaccination, there were variations in their views. Some felt strongly that costs associated with the vaccine was a barrier regardless of the amount. Others, especially participants from the schools in Western Oslo and Viken where the majority were involved in the russ celebration, assumed their parents would cover the cost. For these participants, cost was only a barrier if they themselves had to pay.

#### “If it had been important, we would have received it for free”

A majority of the adolescents emphasized viewing vaccines as less important if they were required to pay for it. Their experiences from the COVID-19 pandemic, when vaccination was provided free to all, seemed to have impacted their view of cost of vaccination and they seemed in agreement that if a vaccine is important, it should be free. One female said:

*«You got the COVID-vaccine for free because it was something that protected not only you*,* but those around you as well. And it’s like that*,* it was important*,* so everyone got it for free and the same with the vaccines we got earlier in primary school [childhood vaccinations]. We got them for free*,* so then you might think that if the health nurse talks about it and says ‘you have to pay $40 for it*,* but you can take it at school’…I would have been like ‘it is probably not that important.’ Because if it had been important*,* then we would have received it for free like all the other vaccines we have received in the past.»* (Female, high school Eastern Oslo)

Many of the adolescents emphasized feeling that when a vaccine is provided for free, the government has prioritized the vaccine by choosing to pay for it. Whether or not the vaccine was paid for by the government was seen as proof of the vaccine’s importance and if they should get it, as illustrated by this adolescent:

*«In a way*,* I don’t think it’s the price itself or the money that matters*,* I think it’s more what it says about how important the vaccine is. Because if it’s a vaccine that costs $500*,* you think ‘okay*,* the government doesn’t want me to have that anyway’ or ‘it’s not important because…’. And it can certainly be the same for the parents*,* that they think that ‘then it’s not that important’.» (*Female, high school Viken)

There seemed to be an agreement, across all focus groups and schools, that when the vaccine costs money the vaccine seems less important because fewer are able to get it. If it’s expensive, it sends the message that not everyone needs this vaccine, it’s optional. The cheaper it is, the more available it becomes. And when the vaccine is completely free, it’s made available for everyone, and more people will be able to get vaccinated.

### Theme 3: Location and guidance

In addition to access to information and free vaccination, the adolescents highlighted several additional factors that influenced their perception of vaccine accessibility, most of which related to how vaccination was organized within the school setting. Based on their previous experiences with vaccines provided through the NIP, the participants described a strong association between vaccination and the school setting.

#### “We are used to vaccines and information being distributed at school”

The majority of the adolescents we talked to, regardless of high school, told us that they had received little, if any, information about IMD and the vaccine from their high school. Most emphasized getting information from older students, friends or family. When asked how they would prefer learning about meningococcal vaccine and disease, all agreed that they want to be educated and not having to find information on their own. Many of the adolescents mentioned that they would prefer to learn about the vaccine at school and said that this would be a good place to reach them, as they are always there. It would ensure that all students would get the information and involvement from the school would make the vaccine seem more important and relevant for adolescents like themselves. Furthermore, most seemed to prefer that someone would physically come to their class and talk to them about IMD and meningococcal vaccine. They highlighted the importance of having the school nurse or someone with a medical background who would be able to answer questions, as illustrated by this adolescent:

*«It’s very efficient to have someone working with it talking about it directly to you*,* not having to read about it or get a message about it. Perhaps health care workers could visit each class and talk about it*,* explain what would be negative aspects of getting meningitis and what the vaccine protects against*,* how it works and so on. And maybe the school could have a written reminder at the school’s website so that you remember.» (*Female, high school Western Oslo)

In addition, regardless of involvement in russ celebration or school location, several of the adolescents wanted to receive information at an early stage, such as at the beginning of high school, and not just prior to vaccination in their senior year. They viewed it as necessary to have time to understand the information received, in order to make a proper decision, but also to be more aware of risk-behavior:

*«It really depends on the school and on an individual basis. But I also think that just being aware of it [the vaccine] and knowing something about it*,* because I had never heard about it before. That you get to know things a little earlier*,* like in the last year of middle school and thus are more aware of it.»* (Female, high school Eastern Oslo)

Furthermore, those who were not planning on participating in the russ celebration emphasized the urgency to communicate the risk of IMD and the importance of the vaccine to all adolescents instead of focusing on those highly involved in the russ celebration. They highlighted that it is especially important to inform parents about disease transmission and that this is a vaccine that is important for all youth, not only those partying and drinking. As illustrated by this statement:

*«What I mean is that if my mom had read the information with me and seen ‘russ celebration’*,* my mother would probably have been like ‘yes*,* but you are not going to do that*,* so it probably doesn’t impact you as much as it does other youth’.» (*Female, high school Eastern Oslo)

Several of the focus groups discussed talking with parents about vaccination and mentioned that it would be useful if their parents would receive more information about the vaccine to enable discussions with their parents. They explained that when they turn 18 and become legal adults, the schools will no longer send any information to their parents.

The participants varied in terms of how much they believed their parents knew about the meningococcal vaccine and disease. Some assumed their parents knew little, if anything, about the MCV4 vaccine and wouldn’t be able to answer any questions they might have, while others described how their parents had influenced their choices, such as this student who had been vaccinated prior to our interview:

*«Even though we have turned 18*,* [our] parents have a very big influence on us…it was my mother who encouraged me to get the vaccine. She said something like ‘I’ve booked an appointment for you so you can take it’. I’m happy about that and I think that it’s good that parents are informed because they can inform their child.» (*Female, high school Viken)

Thus, despite being able to consent to their own vaccination, information to both the adolescents themselves and their parents was desired by many. The adolescents also emphasized the importance of being educated by someone with a health background, such as the school nurse, preferably at school where all students would have access to the information.

#### “When they provide the vaccine at school, it’s pretty safe”

The adolescents varied in whether they perceived place of vaccination as a barrier or facilitator to vaccination, with most being willing to travel to a vaccination center if the vaccine was free. However, they all agreed that the preferred place of vaccination was at school, during school hours. This was described as easier because they are already there every day, it would guarantee equal access for all students, and it would make it harder to accidentally forget about a vaccination appointment. Furthermore, many also believed that more people would get vaccinated because they would see their friends get the vaccine at school. Seeing your friends and classmates get the vaccine was described as important because if they didn’t know anyone that was getting the vaccine, this could impact their own vaccination choices:

*«If no one around you gets it*,* it’s not certain that you will bother [getting vaccinated]*,* because then you don’t think it’s that important.» (*Female, high school Viken)

Being offered vaccines along with their classmates was something they were used to, the school setting was described as familiar, and the school was clearly a place where they felt comfortable and safe. Participants from all schools mentioned that getting vaccinated at school would make the vaccine feel safer, such as these adolescents:

*«We are 18 years old*,* but we are still used to vaccines and information being distributed at school. It’s not something you have to seek out for yourself. It would be like in secondary school when the school nurse came and said ‘you have to take this vaccine’ and then you take it.» (*Female, high school Western Oslo)

*«When it happens at school*,* I feel that it’s a bit less serious. I feel like when they provide it at school*,* it’s pretty safe… if that makes sense?»* (Female, high school Western Oslo)

In addition to being a safe place to get vaccines, being offered the vaccine at school seemed to make the choice of vaccination simpler, even among those who viewed the vaccine as less relevant because they were not going to be heavily involved in the graduation celebration, such as this female:

*«But if it’s like… you’re going to [participate in the russ celebration] and you’re not on a bus*,* just going out a couple of times…I probably wouldn’t have bothered to go somewhere to get it. But when it is actually at school*,* I’ll take it because it’s here.» (*Female, high school Eastern Oslo)

However, regardless of where the vaccine is offered, the adolescents all emphasized that without enough information about the vaccine and disease it is challenging to decide whether the MCV4 vaccine is important on an individual basis. As explained by this adolescent:

*«If you get some information about the disease and what the vaccine does*,* you are more likely to take the vaccine.»* (Male, high school Viken)

## Discussion

This is the first study, to our knowledge, to explore adolescents’ knowledge and views of MCV4 vaccination in Norway. The focus groups with the adolescents highlight the importance of awareness, cost, and location and guidance in their decision-making processes. Although many topics were discussed, we find that the combination of these factors and considerations impact the adolescents’ awareness, affordability, and motivation to accept the MCV4 vaccine.

There was great variation in the adolescents’ awareness of IMD and the vaccine. We find that adolescents from schools in Eastern Oslo, who typically participate less in the russ celebration [10], were less likely to have heard about the vaccine or view it as relevant for themselves, compared to adolescents from schools in Western Oslo or Viken. Among the latter group, most were planning on taking part in the graduation celebration and their view of the vaccine as closely connected to the celebration and associated behavior, which was evident from the interviews. Overall, the adolescents who were not planning on being part of the russ celebration, as well as those who were not going to be part of a russ bus, considered the vaccine less relevant. As evident from past research [9, 12] and this study, the vaccine is often associated with the russ celebration characterized by high alcohol consumption and partying. Thus, those who are less involved in the russ celebration might not consider themselves part of the target group for MCV4 vaccine. The only exception to this view of the vaccine was among adolescents who knew of someone who had been infected with IMD. This group expressed having extensive knowledge about the disease and vaccine regardless of planning to partake in the russ celebration. Overall, this supports past studies finding that knowledge about meningococcal vaccine and disease is important for adolescents’ vaccination decision-making [20–23].

Regardless of school location, knowledge level or vaccination status prior to the interviews, the majority of the adolescents in this study stated that they did not receive enough information about the MCV4 vaccine to be able to make an informed decision about whether to get vaccinated. This is similar to previous studies highlighting that many adolescents feel that school-based vaccine education and information available through healthcare providers is inadequate for making an informed decision about vaccination [14]. Indeed, our focus groups revealed that many of the adolescents were participating in activities that put them at risk for IMD prior to their senior year, such as taking part in the russ celebration of older students, sharing bottles on a regular basis, as well as having close contact with others in their age group [12]. For many of the adolescents, such activities started when they entered high school. However, few adolescents had been vaccinated at the time of the interview.

Cost was identified as an important barrier to vaccination among the adolescents. Similar to other studies [35], we found that the majority of the participants were not willing to spend their own money on the vaccine. Instead, the majority of the participants agreed that the vaccine was perceived as less important when it is not publicly funded. This confirms findings from other studies where out-of-pocket cost of government recommended vaccination has been identified as a substantial barrier to uptake [36]. Adolescents who had already paid for the vaccine or who lived in a region where MCV4 vaccination was not publicly funded relied on their parents to cover the cost [14].

Parental advice and support were also important when making a choice of whether to get vaccinated, similar to findings from past studies [20–22, 24]. Despite all participants being older than 16 and thus required to consent to vaccination on their own, many expressed wanting their parents to receive information about IMD and the MCV4 vaccine prior to being offered vaccination. It thus seems of importance to increase not only information about IMD and the vaccine given to adolescents, but also to their parents. Previous studies have found that adolescents may rely on parents when faced with the choice of vaccination [14, 20–22, 24] and that parental knowledge about IMD is important for facilitating MCV4 vaccination [17]. It seems likely that this will further improve the uptake of the MCV4 vaccine as studies have found increased information linked to higher acceptance of meningococcal vaccines [18, 37].

Our study highlights the need to improve the current organization of MCV4 vaccination in Norway to ensure that all adolescents have equal access to vaccination. School-based vaccination is favored by the majority of the participants in the study, which supports past studies finding that location is important for MCV4 vaccination [25, 26], with lower uptake when distance to vaccination location is larger [25]. In addition to affordability and location, increased awareness is key to ensure that all adolescents have knowledge about the vaccine when receiving an offer of vaccination. Previous studies have found that promoting equal access to vaccination for everyone has several benefits, such as reducing the impact on the healthcare system and reducing social disparities [38]. Furthermore, as the MCV4 coverage in Norway has increased over the past 10 years, there has been a five-fold decrease in IMD cases [39], further highlighting the importance of ensuring that the coverage continues to increase across all regions. However, regional disparities in coverage persists and many adolescents are still required to pay for the vaccine themselves. As lack of information and the cost of vaccination has been identified as direct barriers to vaccination among adolescents in this study, the perhaps easiest way to ensure equal access to the vaccine is to implement it into the well-organized and highly trusted NIP [8]. In fact, introducing the MCV4 vaccine to 15-year-olds in a NIP setting has been found to be cost-effective when compared to today’s practice [9] and would ensure that the vaccine is provided in the school setting as favored by adolescents in the current study.

Although not utilized as a framework for the current study, the adolescents reasoning for their MCV4 vaccination decision-making aligns with the adapted 5 A taxonomy often used to understand vaccination uptake [40]. Our findings indicate that most of the adolescents have access to the vaccine, but the cost and thus affordability varied. The level of awareness about the vaccine and IMD differed, which was also true for whether the adolescents would accept the vaccine (and acceptance seemed to be higher among those with higher awareness). Lastly, activation varied, with some adolescents having received a lot of information about the vaccine and upcoming offer of vaccination, while other had never heard about it.

## Conclusion

The current organization of MCV4 vaccination in Norway does not meet the information and accessibility needs identified by the adolescents in this study. The adolescents perceive the meningococcal vaccine as less important compared to other vaccines included in the NIP, largely due to its associated cost and limited access to information about the vaccine and the disease it prevents. The majority of participants reported receiving little, if any, information about the meningococcal vaccine and disease, which made it challenging to make an informed vaccination decision. The adolescents stress how the regional vaccination costs and organization create unequal access to the vaccine and contributes to inequality in health among Norwegian adolescents. The study indicates that when the meningococcal vaccine is not included in the NIP, vaccination practices seem to be more varied, resulting in difference in availability and cost. From the adolescent perspective, implementing the meningococcal vaccination in the NIP would provide both adolescents and their parents with repeated, school-based information about meningococcal disease and vaccination, prior to offering the vaccine free of charge in a school-based setting. Such an approach may promote more informed decision-making and contribute to reducing structural barriers to access, regardless of adolescents’ place of residence or socioeconomic background.

## Supplementary Information

Supplementary Material 1.

## Data Availability

The datasets generated and/or analyzed during the current study are not publicly available due to the data containing information that could compromise research participant privacy/consent but are available from the corresponding author upon reasonable request and approval of principal investigators and the Norwegian Institute of Public Health. The interview guide, survey and codebook are available upon request.

## Abbreviations

HPV: Human papilloma virus
IMD: Invasive meningococcal disease
MCV4: Meningococcal ACWY conjugate vaccine
NIP: National Immunization Program for Children
NIPH: Norwegian Institute of Public Health
WHO: World Health Organization
