# Supplementary material for: ”Why should I pay for this vaccine?” – Norwegian adolescents views on meningococcal vaccination
Source: BMC Public Health. 2026 Apr 30;26:1875. doi: 10.1186/s12889-026-27591-y (PMC13274219; doi:10.1186/s12889-026-27591-y)
Supplement: Supplementary file 1 — Supplementary Material 1. [file 12889_2026_27591_MOESM1_ESM.docx]

**INTERVIEW GUIDE**

**WELCOME**

1. Purpose of the study
2. Information about the interview process
3. Informed consent: explanation and signatures if participants are willing to participate

**VACCINES**

What comes to mind when you hear the word vaccine?

- Do any of you know how vaccines work?
- Can you name any diseases or infections that vaccines protect against?

Do you talk about vaccines with your friends?

**KNOWLEDGE ABOUT MENINGOCOCCAL VACCINE**

What do you know about the meningococcal vaccine?

- Do you know what the meningococcal vaccine protects against?
- Do you know why it is important to get the vaccine for your age group?

Had you heard about the meningococcal vaccine before this interview?

- Where did you get this information from?
- Have you received information that the meningococcal vaccine is related to the “russ” celebration?
- Have you talked about the vaccine at home? With your friends?
- Do you know anyone who has been vaccinated with meningococcal vaccine?

Have you been offered the vaccine at school?

- Do you know if you will be offered it?

What do you know about meningococcal disease? Symptoms/severity/consequences?

- Do you know how it is transmitted?
- Have you heard of anyone who has contracted infectious meningitis?

**VACCINATION CHOICES**

Do you want to take the meningococcal vaccine?

- Why/why not?
- Do you make special considerations about the vaccine in relation to being a “russ”?
- Are there other factors that influence your decision to take/not take the vaccine?
- How important are your parents’ opinions? School nurse? Friends?

Do you think there is any stigma associated with this vaccine?

During the pandemic, there has been a lot of focus on vaccination, has the pandemic changed your view on meningococcal vaccination?

The cost of meningococcal vaccination varies depending on where in Norway you live. If the vaccine was not free for you, would you be willing to pay for it?

- If yes, how much would you be willing to pay?

When teenagers like yourself are offered this vaccine, how important do you think the price is in the decision to take the vaccine?

- Do you think this is a vaccine that parents of teenagers would pay for?

Until now, all the vaccines you have received have been free, do you think the cost of the vaccine makes it less important?

When/what grade do you think the vaccine should be offered?

Where do you think the vaccine should be offered?

**INFORMATION**

If you want more information about the meningococcal vaccine, who would you ask?

Who do you trust when it comes to information about vaccines?

- How do you determine if you can trust the information?

Are there any other thoughts about vaccines and vaccination that you would like to share?

Do any of you have questions before we end the interview?

Thank you very much for participating in our project, we really appreciate it.
